# The effect of gamification-based training on the knowledge, attitudes, and academic achievement of male adolescents in preventing substance and internet addiction

**DOI:** 10.1186/s12909-023-04858-1

**Published:** 2023-11-13

**Authors:** Esmaeel Taghipour, Fatemeh Vizeshfar, Nahid Zarifsanaiey

**Affiliations:** 1 Student Research Committee, School of Nursing and Midwifery, Shiraz University of Medical Sciences, Shiraz, Iran; 2 Community-Based Psychiatric Care Research Center, Department of Nursing, School of Nursing and Midwifery, Shiraz University of Medical Sciences, Shiraz, Iran; 3 Department of E-learning, School of Medicine, Shiraz University of Medical Sciences, Shiraz, Iran

**Keywords:** Adolescent, Gamification, Internet addiction, Prevention, Substance addiction

## Abstract

**Background:**

Preventing addiction through training takes precedence over treatment and plays a crucial role in enhancing the well-being of adolescents. Utilizing inclusive and participatory methods can significantly enhance the effectiveness of education. Numerous studies have demonstrated that gamification, as an interactive and comprehensive approach, has the potential to boost teenagers’ motivation to engage in learning and contributes to better comprehension.

**Aim:**

This study aimed to assess the impact of gamification-based training to prevent substance and internet addiction on the knowledge and attitudes of male adolescents. Additionally, the study examined this educational program’s effects on male adolescents’ academic achievement.

**Methods:**

This study employed a quasi-experimental design with a control group. One hundred fourteen male adolescents were randomly assigned to the intervention or control groups. They completed a pre-intervention questionnaire assessing addiction-related knowledge, attitudes, and academic achievement. Subsequently, the intervention group received the gamification-based drug and internet addiction prevention training. Post-tests were conducted immediately after the training and again one month later for both groups.

**Results:**

Before the intervention, there were no significant differences in knowledge of substance and internet addiction, attitudes toward substances and the Internet, and academic achievement between the intervention and control groups (P > 0.05). However, after the intervention, the intervention group demonstrated significantly higher scores in knowledge of substance and internet addiction, attitudes toward substances and the Internet, and academic achievement compared to the control group (P < 0.001).

**Conclusion:**

The current study highlights the positive impact of gamification-based training on enhancing male adolescents’ knowledge, attitudes, and academic achievement.

**Supplementary Information:**

The online version contains supplementary material available at 10.1186/s12909-023-04858-1.

## Background

During adolescence, judgment and decision-making skills are developing, and the ability to accurately assess risks and make decisions is underdeveloped [1]. As a result, there is a global increase in the prevalence of risky health behaviors among adolescents, such as addiction [2]. Today, addiction primarily refers to excessive use of substances like cigarettes, alcohol, or drugs. However, addiction is not limited to these substances; it also includes other addictive behaviors, such as Internet addiction, which some psychiatrists consider a form of addiction. Internet addiction can lead to behavioral dependence and deserves attention. Addiction is a complex bio-behavioral disorder characterized by a loss of control, compulsive use, dependency, and a strong desire for a particular activity, substance, or food [3].

Adolescence is a pivotal developmental stage marked by profound physical, cognitive, emotional, social, and behavioral transformations. The biological and neural changes during this period render adolescents more susceptible to the initiation of substance abuse, the development of substance use disorders, and the experience of long-lasting and severe adverse effects associated with substance misuse [4].

The scientific definition of substance abuse is a chronic, recurring mental disorder of the brain [5] that results from problematic and illegal use of psychotropic substances [3]. Adolescents often perceive substances differently, which can lead to risky experimentation, particularly in the presence of peers [6]. The initiation of substance abuse is linked to detrimental behaviors, aggression, conduct disorders, and other mental health issues [7]. Furthermore, substance abuse can adversely affect academic performance, lead to school dropout, and cause cognitive impairments in adolescents [8].

International organizations have provided significant statistics to highlight the scope of these issues. According to the Substance Abuse Department of the World Health Organization, 18.4% of people over 15 worldwide report heavy alcohol consumption, 15.2% daily tobacco use, 3.8% cannabis and marijuana consumption, 0.77% amphetamine use, 0.37% opioid consumption, and 0.35% cocaine use [8]. Furthermore, in the United States, studies have shown that 34.1% of adolescents under 16 have their first experience with alcohol, marijuana, and cigarettes [9].

While the Internet serves various global purposes, such as education, business, and recreation, problems arise when its use negatively impacts teenagers’ physical, mental, and social health [10, 11].

Today, alongside substance addiction, there are other behaviors, such as Internet addiction, which psychiatrists believe can lead to behavioral dependence and warrant attention [10]. Among Internet users, adolescents are particularly vulnerable to Internet addiction compared to other age groups [11].

Internet addiction is characterized by the inability to resist the urge to be online, difficulty spending time offline, and experiences of anxiety and aggression. It can also lead to disruptions in family, friends, and school relationships due to uncontrolled Internet use. Internet dependence shares similar characteristics, including an inability to resist the desire to be online and difficulty engaging in face-to-face interactions. This issue can result in anxiety, aggression, strained family relationships, impaired social connections, and a decline in academic performance [12]. The Internet’s inappropriate use can adversely affect adolescents’ mental health, social life, physical well-being, and educational success [11].

A meta-analysis of data from 31 global studies in seven regions found that the prevalence of Internet addiction in the Middle East is 10.9%, while the lowest prevalence is in North and West Europe at 2.6%. This study also revealed an inverse relationship between the prevalence of Internet addiction and quality of life [13]. In Southeast Asia, studies indicate a variable prevalence of Internet addiction among students, ranging from 0 to 47.4%, with possible prevalence figures ranging from 7.4 to 46.4% [14].

Education is a vital tool in preventing health problems, and interactive multimedia methods, among various media and virtual approaches, have proven to be more effective in enhancing learning [15]. These methods leverage multiple visual and auditory channels, increasing motivation and retention. They offer a suitable and engaging means to educate adolescents about health principles [16].

In contrast, traditional training methods often promote passive learning, neglect individual differences and the specific needs of learners, and fail to address critical thinking and higher-level cognitive skills [17].

In recent years, e-learning techniques, particularly smartphone-based learning, have significantly developed [18]. Additionally, gamification has garnered increased interest as an effective approach. Utilizing participatory methods in preventive education enhances its effectiveness. As an interactive method, gamification can potentially boost adolescents’ motivation to participate in education and improve its effectiveness [19].

One innovative and engaging method for teaching adolescents and exploring the world of electronic multimedia is gamification. Gamification involves applying game-like elements to tasks that may not inherently involve play, turning otherwise dull activities or educational lessons into engaging experiences [18, 19].

Zickermann initially described gamification as a process incorporating game thinking and mechanics to engage users in problem-solving. Later, he refined this definition, stating that gamification involves engaging the audience through the application of game design, behavioral economics, and the best practices in programs [20]. Gamification entails using game elements in non-gaming contexts and has promising applications in addressing global epidemics and chronic diseases [21].

Through gamification, teachers can actively engage their audience with natural stimuli, making education exciting and practical while capturing the audience’s attention, promoting interaction, and enhancing learning [20]. One of the primary goals of gamification is to stimulate motivation and foster cooperative learning, leading to increased knowledge acquisition [14]. Furthermore, gamification can extend its impact beyond learning, teaching various life skills and beliefs [19, 22].

Various studies have demonstrated the positive effects of gamification on academic performance, health promotion, attitudes, and learning behaviors [23–25].

Numerous studies have explored adolescent addiction prevention, each focusing on specific aspects [26, 27, 6, 11]. However, these studies often concentrate on singular dimensions of prevention. In some of these works, researchers emphasized the attitude variable, giving less consideration to knowledge about addiction and its implications on academic achievement, particularly among 13-15-year-olds, a critical age group highly susceptible to addiction influence.

Furthermore, most of these studies relied on traditional educational methods, paying less attention to innovative training techniques. In response to these gaps, this study aimed to assess the impact of gamification-based training on the knowledge and attitude of male adolescents regarding preventing substance and internet addiction. Additionally, the study explored how this educational program affects their academic achievement.

## Materials and methods

### The study environment and population

This study employed a quasi-experimental design with pre-test and post-test measurements, including a control group. The research population consisted of male adolescents aged 13–15 who attended health centers affiliated with Shiraz University of Medical Sciences in 2021.

The authors used MedCalc software version 18 to determine the sample size, with a 95% confidence level, a 5% first type error (α), and an 80% test power (β). After accounting for potential attrition, the sample was 57 individuals per group.

Random sampling was used to select participants from 32 comprehensive health centers in Shiraz. Four centers were randomly chosen. Households with male adolescents aged 13 to 15 were identified, listed, and assigned numbers from each of these centers. Randomly generated numbers were matched to select the intervention group’s sample, and the next adolescent on the list was assigned to the control group. This process continued until 114 participants were included.

Inclusion criteria required the adolescent and their parents to provide informed consent for the adolescent’s participation. Adolescents were also required to have access to a smartphone and be fluent in Persian. Exclusion criteria included a history of educational rejection, smoking or substance abuse, disconnection from the selected comprehensive health centers’ coverage area, and previous participation in addiction prevention training classes.

### Method of study

Initially, participants were contacted by phone. The purpose of the study, the methodology, and the commitment to information confidentiality were explained to motivate honest responses from the adolescents. In light of COVID-19 conditions, informed consent forms were collected online from the students and their parents.

The authors formed a WhatsApp group to communicate the study’s goals and support participants throughout all stages. Questionnaires were distributed online in a pre-test format.

The intervention group received a mobile application for addiction prevention training, incorporating gamification elements like scoring, badges, rewards, and feedback. The program consisted of questions and answers accompanied by educational images and music. Participants couldn’t revisit previous questions. They earned one point for each correct answer and received a star or badge for every five. Incorrect responses prompted the display of the correct answer. Users could engage with the application as often as desired, aiming to improve their scores and assess their progress compared to previous attempts.

The educational content covered several topics, including the definition of addiction, understanding the concept and risks of addiction, identifying various drugs and their types, recognizing the concept and types of internet addiction, and understanding addiction’s physical, psychological, social, and academic impacts. The content also addressed signs and symptoms of drug and internet addiction in teenagers, activities to prevent addiction and proper internet usage.

The participants’ questions about the content were answered through the WhatsApp group throughout the study. After this initial phase, both groups completed the questionnaire in the immediate post-test and again one month later, all done online. The same educational content was eventually provided to the control group as part of ethical compliance.

### Data collection and statistical analysis

The research instrument included a five-part questionnaire covering demographic information, addiction knowledge, attitudes toward substances and the Internet, and academic achievement.

The Addiction Knowledge Questionnaire was designed by the researchers and consisted of 18 true/false questions, with 10 related to substance addiction and 8 related to internet addiction. The questionnaire’s face and content validity were assessed by 10 faculty members from Shiraz University of Medical Sciences specializing in psychometrics, virtual education, and adolescent health. Quantitative methods were employed to determine the content validity of the addiction knowledge questionnaire.

Each item was rated on a 3-point scale (necessary, useful but not necessary, unnecessary) for content validity. All items had a content validity ratio (CVR) score higher than 0.8, confirming their validity. Waltz and Basel’s Content Validity Index (CVI) was utilized to assess the relevance of questionnaire items. This four-point Likert scale (1 = not relevant, 2 = needs revision, 3 = relevant with minor revision, 4 = very relevant) was applied by experts to each item. CVI scores were computed by dividing the number of experts rated the item as 3 or 4 by the total number of experts. Items were evaluated against these criteria: CVI > 0.79 (suitable), CVI < 0.79 (requires review and modification), and CVI < 0.70 (unacceptable). The results indicated that all items scored higher than 0.99. Thus, each item’s content validity index (CVI) exceeded 0.99.

The questionnaire was administered to 25 teenagers not part of the study group using a test-retest method to assess its reliability. The correlation coefficient for questions related to drug addiction knowledge was 0.847; for internet addiction knowledge questions, it was 0.854; and for the entire questionnaire, it was 0.781.

The Attitude to Substances Questionnaire comprises 25 questions divided into five subscales: tendency toward substances (12 questions), reluctance to participate in prevention efforts (5 questions), incorrect belief in positive substance effects (3 questions), lack of active resistance to substances (3 questions), and incorrect belief in the high prevalence of substance abuse (2 questions). Responses are recorded on a five-point Likert scale. Higher scores on this questionnaire indicate a more positive and potentially dangerous attitude toward substances.

A Cronbach’s alpha coefficient of 0.875 supports the questionnaire’s reliability. The authors conducted an exploratory factor analysis to assess its construct validity, revealing five factors that explain approximately 61% of the total variance [28].

The Attitude to Internet Questionnaire consists of 40 questions organized according to the Internet Attitude Scale (IAS) model, comprising four subscales: feeling of self-efficacy, recognizing the Internet’s usefulness, Internet anxiety, and Internet enjoyment. Responses are recorded on a five-point Likert scale, with each subscale containing 10 questions. Higher scores on this questionnaire indicate a more favorable attitude toward the Internet.

The questionnaire’s reliability was evaluated using internal consistency and test-retest, resulting in an internal consistency coefficient of 0.93 and a retesting coefficient of 0.83. Confirmatory factor analysis was employed to assess construct validity. The criterion and Bartlett’s test (P > 0.889) confirmed sample size sufficiency, making factor analysis appropriate for the data. Subsequently, confirmatory factor analysis was conducted using software and orthogonal rotation [29].

Herman’s Academic Advancement Questionnaire comprises 29 incomplete sentences with four response options each. Scores on this questionnaire range from 29 to 116, with higher scores indicating better academic achievement. The questionnaire demonstrates good reliability, with a Cronbach’s alpha coefficient of 0.84.

The authors utilized factor analysis based on the correlation matrix to assess the questionnaire’s construct validity. The analysis revealed general factors such as self-confidence, perseverance, career choice, foresight, and hard work, which collectively explained 40.62% of the overall variance [30].

The data were analyzed using Statistical Software (SPSS) version 18, employing descriptive and analytical statistics. A significance level of P < 0.05 was used for all tests. The normality of the data was assessed using the Kolmogorov-Smirnov test on three occasions in both groups. As the data did not meet the criteria for normal distribution, non-parametric tests were employed. The tests utilized included the Mann-Whitney test and repeated measures analysis using the Friedman test.

## Results

The study involved 114 adolescents aged 13 to 15, with an equal distribution of 50% in the intervention group and 50% in the control group. The subjects had a mean age of 14.0 years with a standard deviation of 6.85.

Most adolescents in both groups were the first child in their families (50 individuals, 43.8%). Most (46 individuals, 40.3%) came from families with two children. Furthermore, the parents of most adolescents (111 individuals, 97.3%) reported no history of smoking or substance abuse (Table 1).

**Table 1:** Comparison of the frequency distribution of demographic characteristics of adolescents in the two groups of intervention and control

Demographic variable		control group (57)		Intervention group (57)		Total	P-Value
		Percentage	Number	Percentage	Number		
Age	13	29.8	17	36.8	21	38	0.693
	14	29.8	17	24.6	14	31	
	15	40.4	23	38.6	22	45	
Number of children at home	1	15.8	9	21.1	12	21	0.541
	2	40.4	23	40.4	23	46	
	3	33.3	19	22.8	13	32	
	4 to up	10.5	6	15.8	9	15	
Birth Rank	1	40.4	23	47.4	27	50	0.518
	2	36.8	21	29.8	17	38	
	3	17.5	10	12.3	7	17	
	4 to up	5.3	3	10.5	6	9	
Father’s education	High school	3.5	2	8.8	5	7	0.183
	Diploma	36.8	21	21.1	12	33	
	Associate Degree	15.8	9	24.6	14	23	
	Bachelor’s degree	43.9	25	45.6	26	51	
Mother’s education	High school	22.8	13	21.1	12	25	0.838
	Diploma	38.6	22	36.8	21	43	
	Associate Degree	15.8	9	12.3	7	16	
	Bachelor’s degree	22.8	13	29.8	17	30	
Father’s job	Freelance	57.9	33	59.6	34	67	0.602
	Employee	40.4	23	40.4	23	46	
	jobless	1.8	1	0	0	1	
Mother’s job	Freelance	10.5	6	10.5	6	12	0.136
	Employee	3.5	2	14	8	10	
	housewife	86	49	75.5	43	92	
Family income	Less than 2 million tomans	1.8	1	0	0	1	0.468
	2–4 million tomans	26.3	15	21.1	12	27	
	More than 4 million tomans	71.9	41	78.9	45	86	

Before the training, no significant differences existed between the intervention and control groups regarding knowledge scores on substance and internet addiction, total attitude scores towards substances and the Internet, their subscales, and academic achievement (P > 0.05).

However, following gamification-based training, there was a significant increase in the mean knowledge scores on substance and internet addiction in the intervention group (P < 0.001). This increase in addiction knowledge persisted immediately after training and one month later (P < 0.001) (Table 2).

**Table 2:** Comparison of mean scores of (substance abuse and Internet) knowledge in and between the intervention and control groups

Variable		control group	Intervention group	P-Value
		M ± SD	M ± SD	
Knowledge of substance abuse	Pre test	4.35 ± 2.20	4.40 ± 2.20	0.663
	Post test	4.82 ± 2.03	9.04 ± 1.21	0.000
	1 month PT	4.35 ± 1.84	8.46 ± 1.33	0.000
	P-Value	0.152	0.000	-
Internet addiction knowledge	Pre test	3.30 ± 1.94	3.25 ± 1.73	0.954
	Post test	2.95 ± 1.46	7.42 ± 0.86	0.000
	1 month PT	3.26 ± 1.45	6.88 ± 0.88	0.000
	P-Value	0.556	0.000	-

After the addiction prevention training, significant differences were observed in various aspects for the intervention group compared to the control group. Specifically, the intervention group showed statistically significant improvements in their total attitude scores toward substances and their subscales, total attitude scores toward the Internet and its subscales, and academic achievement (P < 0.001).

Furthermore, within the intervention group, the mean score for the tendency toward substance abuse showed a significant increase immediately after the intervention, and this increase was sustained one month later. The intervention group also demonstrated significant improvements in the mean scores for reluctance to actively participate in prevention, incorrect belief in the positive effects of substances, lack of active resistance to substance abuse, and incorrect belief in the high prevalence of substance abuse.

Immediately and one month after the intervention, the intervention group demonstrated statistically significant differences compared to the control group, with more pronounced changes. Specifically, the intervention group’s attitude toward substances significantly decreased, indicating the development of a more negative attitude toward substances (P < 0.001).

In contrast, the intervention group’s average score for their attitude toward the Internet significantly increased immediately and one month after the intervention, suggesting a more positive attitude. Academic achievement also saw an increase only in the intervention group immediately and one month after training, highlighting the positive impact of the training on individuals’ Internet attitudes and academic performance among adolescents in the intervention group (Tables 3 and 4).

**Table 3:** Comparison of mean attitudes toward drugs in and between the intervention and control groups

Variable		control group	Intervention group	P-Value
		M ± SD	M ± SD	
Tendency to substances	Pre test	38.82 ± 8.17	38.23 ± 7.98	0.565
	Post test	40.11 ± 7.87	16.02 ± 3.92	0.000
	1 month PT	39.96 ± 7.30	17.00 ± 4.03	0.000
	P-Value	0.03	0.000	-
unwillingness to active participation in prevention	Pre test	14.49 ± 4.57	14.56 ± 4.71	0.977
	Post test	15.04 ± 4.23	6.04 ± 1.48	0.000
	1 month PT	15.02 ± 3.85	6.81 ± 1.34	0.000
	P-Value	0.223	0.000	-
incorrect belief in positive effects of substances	Pre test	9.95 ± 2.37	9.95 ± 2.58	0.708
	Post test	10.40 ± 2.35	3.65 ± 1.18	0.000
	1 month PT	10.35 ± 2.29	4.04 ± 1.32	0.000
	P-Value	0.298	0.000	-
lack of active encountering with substances	Pre test	10.23 ± 2.39	10.42 ± 1.71	0.905
	Post test	10.42 ± 2.07	4.53 ± 1.36	0.000
	1 month PT	10.44 ± 2.14	4.89 ± 1.16	0.000
	P-Value	0.496	0.000	-
**the incorrect belief in the high prevalence of substance abuse**	Pre test	8.05 ± 1.55	7.91 ± 1.49	0.516
	Post test	8.18 ± 1.56	2.95 ± 1.10	0.000
	1 month PT	8.28 ± 1.52	2.84 ± 1.03	0.000
	P-Value	0.723	0.000	-
Total score of substance attitude	Pre test	81.53 ± 16.12	81.07 ± 15.97	0.834
	Post test	84.14 ± 15.83	33.21 ± 7.59	0.000
	1 month PT	84.05 ± 15.06	35.58 ± 7.78	0.000
	P-Value	0.102	0.000	-

**Table 4:** Comparison of the mean score of Internet attitude and academic achievement score in and between the intervention and control groups

Variable		control group	Intervention group	P-Value
		M ± SD	M ± SD	
Feeling of self-efficacy	Pre test	28.11 ± 5.99	27.58 ± 5.38	0.622
	Post test	26.81 ± 4.48	39.68 ± 2.40	0.000
	1 month PT	27.30 ± 6.15	39.79 ± 2.64	0.000
	P-Value	0.182	0.000	-
recognizing internet to be useful	Pre test	31.67 ± 5.97	31.61 ± 5.38	0.802
	Post test	30.79 ± 4.26	47.70 ± 3.16	0.000
	1 month PT	31.02 ± 5.03	45.63 ± 2.69	0.000
	P-Value	0.528	0.000	-
Internet anxiety	Pre test	25.72 ± 6.10	24.91 ± 5.22	0.618
	Post test	23.75 ± 4.36	46.44 ± 3.54	0.000
	1 month PT	24.65 ± 6.01	45.37 ± 3.06	0.000
	P-Value	0.078	0.000	-
enjoyment of the Internet	Pre test	29.54 ± 6.73	28.67 ± 5.96	0.506
	Post test	27.19 ± 5.31	46.51 ± 2.89	0.000
	1 month PT	28.56 ± 6.51	45.49 ± 2.68	0.000
	P-Value	0.041	0.000	-
Total score of internet attitude	Pre test	115.04 ± 22.74	112.77 ± 20.10	0.768
	Post test	109.46 ± 17.52	180.33 ± 9.79	0.000
	1 month PT	111.53 ± 21.86	176.28 ± 6.43	0.000
	P-Value	0.142	0.000	-
Academic Achievement	Pre test	70.40 ± 15.88	70.05 ± 17.43	0.832
	Post test	68.07 ± 16.37	96.49 ± 8.61	0.000
	1 month PT	69.00 ± 15.69	91.19 ± 5.48	0.000
	P-Value	0.179	0.000	-

## Discussion

Table 1 shows no significant differences in demographic characteristics between the intervention and control groups. This finding indicates the adequacy of the sampling process in both groups and enhances the inferential power when comparing the groups on the main study variables.

The results of this study highlight the positive impact of gamification on improving students’ knowledge, attitude, and academic achievement. Gamification enhances the teaching process, making it more efficient and effective. This method can find applications in medical sciences and health education, promoting healthier lifestyles. A systematic review also supports the validity of gamification in teaching health-related programs and enhancing academic achievement among students. Its increased motivation is a compelling reason to consider gamification techniques in educational processes [23].

A study in Korea demonstrated the positive impact of gamification in improving learning outcomes, particularly when game elements are integrated, which fosters positive learning attitudes and behaviors while reducing the risk of internet addiction [24].

Gamification is a valuable learning strategy that can potentially enhance adolescent educational performance [31]. Additionally, research suggests that the use of the Healthy Internet Use Program can help reduce the rate of Internet addiction among adolescents [9].

Furthermore, studies by Gabaron et al. (2013) and Harona et al. (2018) concluded that gamification-based training is an accessible and effective intervention model for adolescents compared to traditional methods [32, 33]. Chau et al. (2019) found that educational interventions using gamification led to increased knowledge among students, reduced symptoms of internet disruption, and decreased number of students at risk of this disorder, resulting in improved emotional well-being [34].

The study’s findings, aligned with the research hypotheses, revealed that the intervention group demonstrated a significant increase in addiction knowledge immediately and one month after the training intervention. Additionally, the intervention group showed significantly improved attitudes toward substances and their various subscales, reaching a favorable level immediately and one month after the training. Moreover, the attitude toward the Internet and academic achievement increased compared to before the intervention and achieved favorable values.

These changes underscore the effectiveness of educational intervention in enhancing adolescent knowledge, fostering a negative attitude towards substance use, promoting a positive attitude towards the Internet, and increasing motivation for academic achievement.

These changes in the intervention group’s attitude towards the Internet following the educational intervention highlight the positive impact of the intervention in fostering a more positive and responsible attitude among adolescents. It’s worth noting that a significant and negative correlation exists between internet addiction and adolescents’ academic achievement, emphasizing the importance of educational efforts to improve adolescents’ motivation and academic performance compared to pre-intervention levels.

Evans et al. (2020) indicated that participation in substance abuse prevention programs improved knowledge, self-efficacy, and safer attitudes among students [6]. Similarly, Mun et al. (2015) found positive effects of an integrated Internet addiction program for elementary students at risk of internet addiction [31].

Joo et al. (2010) assessed the impact of a training program focused on empowerment against addiction, internet games, and stress among junior high school students. They reported higher empowerment scores and lower addiction and stress scores among students in the intervention group after an empowering, addiction prevention, and stress management program [35].

Furthermore, Haruna et al. (2018) demonstrated that gamification-based education in STD prevention programs increased knowledge, improved attitudes, and enhanced student motivation [33].

These findings align with numerous other studies highlighting the positive effects of education on knowledge of addiction, attitudes towards substances and the Internet, and the promotion of academic achievement [33–37, 11].

## Conclusion

This study demonstrated that gamification-based addiction prevention training is an engaging and effective method for enhancing knowledge, improving attitudes, and boosting adolescent academic achievement. Prevention training during adolescence is not only cost-effective but also superior to treatment.

As a recommendation for policymakers and social and health planners, the authors propose using gamification-based training as an attractive, valuable, and cost-effective approach to preventing adolescent addiction.

This study, however, has some limitations. The follow-up duration was short; further investigation is needed to assess the intervention’s long-term effects. Cultural considerations limited the inclusion of both genders and future research should aim to conduct comparative studies with reduced cultural barriers, including both male and female participants.

### Electronic supplementary material

Below is the link to the electronic supplementary material.

Supplementary Material 1

Supplementary Material 2

## Data Availability

Upon request from the first author, data is available. For inquiries related to data access, please contact essitaghipour1997@gmail.com.
