# Supplementary material for: The effect of gamification-based training on the knowledge, attitudes, and academic achievement of male adolescents in preventing substance and internet addiction
Source: BMC Med Educ. 2023 Nov 13;23:860. doi: 10.1186/s12909-023-04858-1 (PMC10641928; doi:10.1186/s12909-023-04858-1)
Supplement: Supplementary file 1 — Supplementary Material 1 [file 12909_2023_4858_MOESM1_ESM.docx]

**Demographic information questionnaire**

| * | questions |  |
| --- | --- | --- |
| 1 | Age( years) | 13 ⃝ 14 ⃝ 15 ⃝ |
| 2 | Number of children | 1⃝ 2⃝ 3⃝ 4 and more⃝ |
| 3 | Birth order (How many children are there?) | 1 ⃝ 2 ⃝ 3 ⃝ Fourth and above ⃝ |
| 4 | Father's education | High school ⃝ Diploma⃝ Associate Degree ⃝ Bachelor's degree and higher ⃝ |
| 5 | Mother's education | High school ⃝ Diploma⃝ Associate Degree ⃝ Bachelor's degree and higher ⃝ |
| 6 | Father's job | self-employment ⃝ Employee⃝ Unemployed⃝ |
| 7 | Mothers Job | self-employment ⃝ Employee ⃝ housewife ⃝ |
| 8 | family income( Rial) | less than 20 M⃝ 2-4M ⃝ more than 4 M ⃝ |
| 9 | Hx of substance abuse | Yes⃝ No⃝ |
| 10 | Addiction in member of family | Yes⃝ No⃝ |
| 11 | I spend a lot of my time daily using the Internet and virtual space | Yes⃝ No⃝ |

**Drug and internet addiction knowledge questionnaire**

| 1 | Smoking causes physical and behavioral dependence | correct ⃝ Wrong⃝ don’t know⃝ |
| --- | --- | --- |
| 2 | Drug addiction is not a chronic and social disease | correct ⃝ wrong⃝ don’t know⃝ |
| 3 | Heroin and glass drug addictive and hallucinogenic substances. | correct ⃝ wrong⃝ don’t know⃝ |
| 4 | Cigarettes and marijuana are from the same family of drugs. (They have similar effects on the user) | correct ⃝ wrong⃝ don’t know⃝ |
| 5 | Addiction causes a series of uncontrollable behaviors in a person (such as aggression and frequent misbehavior). | correct ⃝ wrong⃝ don’t know⃝ |
| 6 | Glue and gasoline can be addictive. | correct ⃝ wrong⃝ don’t know⃝ |
| 7 | Opium use increases sexual desire and power in a person | correct ⃝ wrong⃝ don’t know |
| 8 | Marijuana consumption causes poor memory and learning . | correct ⃝ wrong⃝ don’t know⃝ |
| 9 | Drug use increases true self-confidence and intellectual maturity in a person | correct ⃝ wrong⃝ don’t know⃝ |
| 10 | Long-term use of marijuana and hashish creates a sense of peace and happiness in life | correct ⃝ wrong⃝ don’t know⃝ |
| 11 | Using the Internet only 10 hours a week is considered Internet addiction in teenagers | correct ⃝ wrong⃝ don’t know⃝ |
| 12 | Internet addiction has physical consequences for teenagers. | correct ⃝ wrong⃝ don’t know⃝ |
| 13 | Playing online games for more than 2-3 hours a day is a kind of internet addiction . | correct ⃝ wrong⃝ don’t know⃝ |
| 14 | online Shopping more than usual is not an addiction to the internet . | correct⃝ wrong⃝ don’t know⃝ |
| 15 | A person addicted to the Internet has a proper and detailed plan for her free time . | correct ⃝ wrong⃝ don’t know⃝ |
| 16 | feeling guilty after spending many hours on the Internet is one of the psychological symptoms of Internet addiction. | correct ⃝ wrong⃝ don’t know⃝ |
| 17 | Insomnia and irregular and unhealthy eating habits can be physical symptoms of Internet addiction | correct ⃝ wrong⃝ don’t know⃝ |
| 18 | Reading a book before going to sleep helps to prevent internet addiction. | correct  ⃝  wrong⃝ don’t know⃝ |

**Substance abuse attitude questionnaire**

| 1 | I enjoy hanging out with people my age who use drugs | Completely agree⃝ Somewhat agree⃝ I have no opinion⃝ Somewhat disagree⃝ Completely disagree⃝ |
| --- | --- | --- |
| 2 | Consumption of some substances makes a person lively and funny | Completely agree⃝ Somewhat agree⃝ I have no opinion⃝ Somewhat disagree⃝ Completely disagree⃝ |
| 3 | There is nothing wrong with taking drugs for fun. | Completely agree⃝ Somewhat agree⃝ I have no opinion⃝ Somewhat disagree⃝ Completely disagree⃝ |
| 4 | Taking drugs occasionally is considered a form of harmless or less harmful entertainment . | Completely agree⃝ Somewhat agree⃝ I have no opinion⃝ Somewhat disagree⃝ Completely disagree⃝ |
| 5 | would like to try one of the substance | Completely agree⃝ Somewhat agree⃝ I have no opinion⃝ Somewhat disagree⃝ Completely disagree⃝ |
| 6 | Even if drug use is harmful, it is only for the user and does not harm the society . | Completely agree⃝ Somewhat agree⃝ I have no opinion⃝ Somewhat disagree⃝ Completely disagree⃝ |
| 7 | If one of my peers doesn't use drugs, they consider her a child and un modern | Completely agree⃝ Somewhat agree⃝ I have no opinion⃝ Somewhat disagree⃝ Completely disagree⃝ |
| 8 | *Only indiscriminate and excessive consumption of substances is harmful .* | Completely agree⃝ Somewhat agree⃝ I have no opinion⃝ Somewhat disagree⃝ Completely disagree⃝ |
| 9 | *Drug use increases a person's tolerance to problems .* | Completely agree⃝ Somewhat agree⃝ I have no opinion⃝ Somewhat disagree⃝ Completely disagree⃝ |
| 10 | Drug consumption increases a person's artistic abilities . | Completely agree⃝ Somewhat agree⃝ I have no opinion⃝ Somewhat disagree⃝ Completely disagree⃝ |
| 11 | Because of characterof this age, anyone may use drugs once or twice during adolescence and puberty and not become an addict . | Completely agree⃝ Somewhat agree⃝ I have no opinion⃝ Somewhat disagree⃝ Completely disagree⃝ |
| 12 | *Substance use is beneficial for the elderly* | Completely agree⃝ Somewhat agree⃝ I have no opinion⃝ Somewhat disagree⃝ Completely disagree⃝ |
| 13 | *If a non-governmental group has a program to reduce addiction where I live, work or study, I will try to participate in it.* | Completely agree⃝ Somewhat agree⃝ I have no opinion⃝ Somewhat disagree⃝ Completely disagree⃝ |
| 14 | *If there is going to be an educational material prepared at work or school about successful methods of addiction prevention, I will try to help* | Completely agree⃝ Somewhat agree⃝ I have no opinion⃝ Somewhat disagree⃝ Completely disagree⃝ |
| 15 | *If the police have a plan to reduce the number of drug addicts where I live, work or study, I will try to help them* | Completely agree⃝ Somewhat agree⃝ I have no opinion⃝ Somewhat disagree⃝ Completely disagree⃝ |
| 16 | *If a government organization invites young people to cooperate to reduce addiction, I will try to cooperate with them.* | Completely agree⃝ Somewhat agree⃝ I have no opinion⃝ Somewhat disagree⃝ Completely disagree⃝ |
| 17 | *If I find that a book has been published about successful prevention methods for young people, I try to get it and read it. .* | Completely agree⃝ Somewhat agree⃝ I have no opinion⃝ Somewhat disagree⃝ Completely disagree⃝ |
| 18 | *Taking some substances is a good way to treat some diseases.* | Completely agree⃝ Somewhat agree⃝ I have no opinion⃝ Somewhat disagree⃝ Completely disagree⃝ |
| 19 | *Taking drugs is a good way to reduce pain* | Completely agree⃝ Somewhat agree⃝ I have no opinion⃝ Somewhat disagree⃝ Completely disagree⃝ |
| 20 | *Some substances increase the physical strength of people .* | Completely agree⃝ Somewhat agree⃝ I have no opinion⃝ Somewhat disagree⃝ Completely disagree⃝ |
| 21 | *If I notice that they are using drugs at a party, I will leave immediately.* | Completely agree⃝ Somewhat agree⃝ I have no opinion⃝ Somewhat disagree⃝ Completely disagree⃝ |
| 22 | *I will not buy from a shop whose seller is an addict.* | Completely agree⃝ Somewhat agree⃝ I have no opinion⃝ Somewhat disagree⃝ Completely disagree⃝ |
| 23 | *don't invite a friend who uses drugs to my parties.* | Completely agree⃝ Somewhat agree⃝ I have no opinion⃝ Somewhat disagree⃝ Completely disagree⃝ |
| 24 | *Many people use drugs in their teens* | Completely agree⃝ Somewhat agree⃝ I have no opinion⃝ Somewhat disagree⃝ Completely disagree⃝ |
| 25 | *Many people in society use drugs* | Completely agree⃝ Somewhat agree⃝ I have no opinion⃝ Somewhat disagree⃝ Completely disagree⃝ |

**Internet Attitude Questionnaire**

| 1 | Working with the Internet is not boring for me | Completely agree⃝ Somewhat agree⃝ I have no opinion⃝ Somewhat disagree⃝ Completely disagree⃝ |
| --- | --- | --- |
| 2 | The Internet provides me with more new information than printed sources (books, newspapers, magazines, etc.). | Completely agree⃝ Somewhat agree⃝ I have no opinion⃝ Somewhat disagree⃝ Completely disagree⃝ |
| 3 | don't feel nervous when using the internet | Completely agree⃝ Somewhat agree⃝ I have no opinion⃝ Somewhat disagree⃝ Completely disagree⃝ |
| 4 | I am confident in the ability to send electronic messages (E-mail) to others . | Completely agree⃝ Somewhat agree⃝ I have no opinion⃝ Somewhat disagree⃝ Completely disagree⃝ |
| 5 | I like communicating with others through the Internet | Completely agree⃝ Somewhat agree⃝ I have no opinion⃝ Somewhat disagree⃝ Completely disagree⃝⃝ |
| 6 | Internet has provided suitable tools (Chat, E-mail, etc.) to communicate with others. | Completely agree⃝ Somewhat agree⃝ I have no opinion⃝ Somewhat disagree⃝ Completely disagree⃝⃝ |
| 7 | Whenever I talk to others about the Internet, I am comfortable (I am calm). | Completely agree⃝ Somewhat agree⃝ I have no opinion⃝ Somewhat disagree⃝ Completely disagree⃝ |
| 8 | I'm sure I can use a web browser . | Completely agree⃝ Somewhat agree⃝ I have no opinion⃝ Somewhat disagree⃝ Completely disagree⃝⃝ |
| 9 | I enjoy searching for information on the Internet | Completely agree⃝ Somewhat agree⃝ I have no opinion⃝ Somewhat disagree⃝ Completely disagree⃝ |
| 10 | Internet has become a necessary tool in my daily life | Completely agree⃝ Somewhat agree⃝ I have no opinion⃝ Somewhat disagree⃝ Completely disagree⃝ |
| 11 | When working with the Internet, I feel relaxed (I am relaxed). | Completely agree⃝ Somewhat agree⃝ I have no opinion⃝ Somewhat disagree⃝ Completely disagree⃝ |
| 12 | I am confident that I have the ability to discuss topics with others over the Internet. | Completely agree⃝ Somewhat agree⃝ I have no opinion⃝ Somewhat disagree⃝ Completely disagree⃝⃝ |
| 13 | Because of my interest in the Internet, I can spend a longer time on the Internet | Completely agree⃝ Somewhat agree⃝ I have no opinion⃝ Somewhat disagree⃝ Completely disagree⃝ |
| 14 | I can do my work faster by using the internet | Completely agree⃝ Somewhat agree⃝ I have no opinion⃝ Somewhat disagree⃝ Completely disagree⃝ |
| 15 | I don't think searching the internet is scary . | Completely agree⃝ Somewhat agree⃝ I have no opinion⃝ Somewhat disagree⃝ Completely disagree⃝ |
| 16 | I am sure that I can forward a new electronic message to others. | Completely agree⃝ Somewhat agree⃝ I have no opinion⃝ Somewhat disagree⃝ Completely disagree⃝⃝ |
| 17 | I like working with the Internet | Completely agree⃝ Somewhat agree⃝ I have no opinion⃝ Somewhat disagree⃝ Completely disagree⃝ |
| 18 | It is easy to access (find) useful information on the Internet. | Completely agree⃝ Somewhat agree⃝ I have no opinion⃝ Somewhat disagree⃝ Completely disagree⃝ |
| 19 | I have never felt a failure from working with the Internet. | Completely agree⃝ Somewhat agree⃝ I have no opinion⃝ Somewhat disagree⃝ Completely disagree⃝⃝ |
| 20 | I'm sure I can find useful websites. | Completely agree⃝ Somewhat agree⃝ I have no opinion⃝ Somewhat disagree⃝ Completely disagree⃝⃝ |
| 21 | I prefer to read the information directly from the Internet instead of copying it to the computer hardware. | Completely agree⃝ Somewhat agree⃝ I have no opinion⃝ Somewhat disagree⃝ Completely disagree⃝⃝ |
| 22 | Using the Internet to do things like paying tax forms, registering for school, completing the employment form, etc. is useful (it is affordable). | Completely agree⃝ Somewhat agree⃝ I have no opinion⃝ Somewhat disagree⃝ Completely disagree⃝⃝ |
| 23 | The Internet does not threaten me. | Completely agree⃝ Somewhat agree⃝ I have no opinion⃝ Somewhat disagree⃝ Completely disagree⃝⃝ |
| 24 | am sure that I have the ability to communicate with others through the Internet. | Completely agree⃝ Somewhat agree⃝ I have no opinion⃝ Somewhat disagree⃝ Completely disagree⃝ |
| 25 | The use of Internet search engines is interesting for me. | Completely agree⃝ Somewhat agree⃝ I have no opinion⃝ Somewhat disagree⃝ Completely disagree⃝ |
| 26 | Spending time on the Internet is valuable | Completely agree⃝ Somewhat agree⃝ I have no opinion⃝ Somewhat disagree⃝ Completely disagree⃝ |
| 27 | don't feel anxious when working with the Internet. | Completely agree⃝ Somewhat agree⃝ I have no opinion⃝ Somewhat disagree⃝ Completely disagree⃝ |
| 28 | I am sure that I have the ability to use search engines (Google, Yahoo). | Completely agree⃝ Somewhat agree⃝ I have no opinion⃝ Somewhat disagree⃝ Completely disagree⃝ |
| 29 | Graphic images and communication of internet sites are interesting for me | Completely agree⃝ Somewhat agree⃝ I have no opinion⃝ Somewhat disagree⃝ Completely disagree⃝⃝ |
| 30 | Internet can help us to get information in a satisfactory way | Completely agree⃝ Somewhat agree⃝ I have no opinion⃝ Somewhat disagree⃝ Completely disagree⃝ |
| 31 | Internet does not make me uncomfortable. | Completely agree⃝ Somewhat agree⃝ I have no opinion⃝ Somewhat disagree⃝ Completely disagree⃝ |
| 32 | I am sure that I have the ability to use standard programs related to creating personal sites and home pages such as (HTML). | Completely agree⃝ Somewhat agree⃝ I have no opinion⃝ Somewhat disagree⃝ Completely disagree⃝ |
| 33 | Whenever I start working with the Internet, I tend to continue it as long as I can. | Completely agree⃝ Somewhat agree⃝ I have no opinion⃝ Somewhat disagree⃝ Completely disagree⃝⃝ |
| 34 | The first option for research and search for me is the Internet | Completely agree⃝ Somewhat agree⃝ I have no opinion⃝ Somewhat disagree⃝ Completely disagree⃝⃝ |
| 35 | Dealing with internet search engines does not bother me. | Completely agree⃝ Somewhat agree⃝ I have no opinion⃝ Somewhat disagree⃝ Completely disagree⃝ |
| 36 | I am sure that I have the ability to use software related to creating personal websites (such as HTML, etc.). | Completely agree⃝ Somewhat agree⃝ I have no opinion⃝ Somewhat disagree⃝ Completely disagree⃝ |
| 37 | For research, I prefer the internet to the library. | Completely agree⃝ Somewhat agree⃝ I have no opinion⃝ Somewhat disagree⃝ Completely disagree⃝ |
| 38 | Internet is useful for people in distant places to contact each other. | Completely agree⃝ Somewhat agree⃝ I have no opinion⃝ Somewhat disagree⃝ Completely disagree⃝ |
| 39 | I feel comfortable communicating with others through the Internet. | Completely agree⃝ Somewhat agree⃝ I have no opinion⃝ Somewhat disagree⃝ Completely disagree⃝ |
| 40 | I am sure that I can find the information I need on the Internet | Completely agree⃝ Somewhat agree⃝ I have no opinion⃝ Somewhat disagree⃝ Completely disagree⃝ |

**Academic Achievement questionnire**

| 1 | Working is something that... | A) I don't like to do it at all. b) I don't like to do it  c) I would like to do it. d) I really like to do it |
| --- | --- | --- |
| 2 | At school, they think that I am a person…. | A) I am very hardworking. b) I am hardworking.  c) I am relatively easy-going. d) I am very easy-going. |
| 3 | Other people think that I... | A) I am very hardworking. b) I am hardworking.  c) I am not hardworking. d) I am not hardworking at all |
| 4 | If we prepare ourselves long in advance to do a task, this work... | A) It is really meaningless. b) It is meaningless.  c) It is acceptance of reality. d) necessary for success. |
| 5 | What I expect from myself when I work…. | A) It is very high. B) is high.  C) is low. d) is too low. |
| 6 | When the teacher teaches in the classroom… | A) I usually use my whole being to do my job in the best way and give a good impression to the teacher.  b) I usually pay a lot of attention to the lessons that are taught.  c) When the teacher is teaching, my thoughts are diverted to other things.  d) I am more interested in things that are not related to school. |
| 7 | usually the amount of work I do... | A) It is much more than the decision I intended to make.  b) It is more than the decision I intended to make.  c) It is less than the decision I intended to make.  d) It is much less than the decision I intended to make. |
| 8 | Whenever I didn't do my homework well and didn't reach my goal... | A) I will do my best to achieve the goal. b) I will try again to reach the goal.  c) I would like to ignore it. d) I usually ignore it. |
| 9 | Persistence in school seems to me .......... | A) It is very unimportant. b) It is relatively unimportant.  c) It is important. d) It is very important. |
| 10 | Start doing homework at home. | A) It takes a lot of effort. b) It requires a lot of effort.  c) It requires little effort. d) It requires very little effort. |
| 11 | When I was in the lower classes, the standards and expectations I had of myself according to the amount of my studies... | A) It was very high. b) It was average.  c) was low. d) It was too low. |
| 12 | If I am called to watch TV or listen to the radio while I am doing my homework, then .......... | a) I always return to work immediately.  b) I take a short break and then go back to work.  c) I always wait a bit before starting work again.  d) I find it very difficult to return to work. |
| 13 | I want to do something that requires a lot of responsibility…. | A) I like it very much. b) I will only do it if they pay good money for it.  c) I think I can't do it. d) I don't like it. |
| 14 | That life that does not need to work at all, in my opinion... | A) It is very pleasant. b) It is pleasant.  c) It is unpleasant. d) It is very unpleasant. |
| 15 | When I was in the lower grades, I thought that achieving a high position in society (getting a position and a position) .......... | A) It is very unimportant. b) It is of little importance.  c) It is very important. d) It is very important. |
| 16 | When I face a problem in doing work... | A) I give up doing it very quickly. b) I quickly give up doing it.  c) I will give up doing it very soon. d) I usually do it. |
| 17 | Generally .......... | A) I am very futuristic. b) I am very futuristic.  c) I futuristic. d) I am not futuristic at all |
| 18 | In my opinion, At school,students who study too much... | a) They are very good people. b) They are good people.  c) They are not good people. d) They are not good people at all |
| 19 | In school, those who have reached very high positions | A) I admire a lot. b) I admire a lot.  c) I do not admire. d) I do not admire at all. |
| 20 | To use the extra pleasures and entertainments of life. | A) I don't have time at all. b) I often don't have time  c) I usually have enough time. d) I always have time. |
| 21 | I am usually a person… | a) I am very busy. b) I am busy.  c) I am not busy d) I am not busy at all. |
| 22 | I can without getting tired for .......... | A) I work for a long time on a particular matter.  b) I work for a long time on a particular matter.  c) I will work on a specific matter for a short period of time.  d) To work on a specific matter for a very short period of time. |
| 23 | When I was in lower grades, having a good relationship with my teachers... | A) It was highly appreciated. b) It was highly appreciated.  c) I don't think it was important. d) It seemed to be completely unimportant. |
| 24 | Children follow their father's career as a director of an institution because... | a) They want to develop the activity of the institution.  b) They are happy because their father is a manager.  c) They can implement their new ideas.  d) They can meet their material needs. |
| 25 | When I was in lower grades, I wanted... | A) I am a very important person. b) To be an important person.  c) Be a relatively important person. d) To be an ordinary person |
| 26 | I put things in order ...... | I like it very much. b) I like A)  c) I don't like much. d) I don't like it at all. |
| 27 | When I start something | A) I never continue it until reaching a successful result.  b) I continue it very little until I reach a successful result.  c) Sometimes I continue it until I reach a successful result.  d) I always continue it until reaching a successful result. |
| 28 | I .......... | A) I am tired most of the time. b) I am often tired.  c) I am very little tired. d) I am not tired at all. |
| 29 | hopping is something that I... | A) I like very much. b) I like  c) I don't like it. d) I don't like it at all. |
